# Detection of SARS-CoV-2 RNA in selected agricultural and food retail environments in Tehran, Iran

**DOI:** 10.3389/fpubh.2022.823061

**Published:** 2022-09-23

**Authors:** Maedeh Rafieepoor, Seyed Reza Mohebbi, Seyed Masoud Hosseini, Mohammad Tanhaei, Mahsa Saeedi Niasar, Shabnam Kazemian, Hamid Asadzadeh Aghdaei, Matthew D. Moore, Mohammad Reza Zali

**Affiliations:** ^1^Basic and Molecular Epidemiology of Gastrointestinal Disorders Research Center, Research Institute for Gastroenterology and Liver Diseases, Shahid Beheshti University of Medical Sciences, Tehran, Iran; ^2^Department of Microbiology and Microbial Biotechnology, Faculty of Life Sciences and Biotechnology, Shahid Beheshti University, Tehran, Iran; ^3^Gastroenterology and Liver Diseases Research Center, Research Institute for Gastroenterology and Liver Diseases, Shahid Beheshti University of Medical Sciences, Tehran, Iran; ^4^Foodborne and Waterborne Diseases Research Center, Research Institute for Gastroenterology and Liver Diseases, Shahid Beheshti University of Medical Sciences, Tehran, Iran; ^5^Department of Food Science, University of Massachusetts, Amherst, MA, United States

**Keywords:** SARS-CoV-2, vegetables, irrigation water, wastewater, food-safety, Iran

## Abstract

The SARS-CoV-2 pandemic has and continues to impose a considerable public health burden. Although not likely foodborne, SARS-CoV-2 transmission has been well documented in agricultural and food retail environments in several countries, with transmission primarily thought to be worker-to-worker or through environmental high touch surfaces. However, the prevalence and degree to which SARS-CoV-2 contamination occurs in such settings in Iran has not been well documented. Furthermore, since SARS-CoV-2 has been observed to be shed in the feces of some infected individuals, wastewater has been utilized as a means of surveilling the occurrence of SARS-CoV-2 in some regions. This study aimed to investigate the presence of SARS-CoV-2 RNA along the food production and retail chain, from wastewater and irrigation water to vegetables in field and sold in retail. From September 2020 to January 2021, vegetables from different agricultural areas of Tehran province (*n* = 35), their irrigated agricultural water (*n* = 8), treated wastewater mixed into irrigated agricultural water (*n* = 8), and vegetables collected from markets in Tehran (*n* = 72) were tested for the presence of SARS-CoV-2 RNA. The vegetable samples were washed with TGBE buffer and concentrated with polyethylene glycol precipitation, while water samples were concentrated by an adsorption-elution method using an electronegative filter. RT-qPCR targeting the SARS-CoV-2 N and RdRp genes was then conducted. SARS-CoV-2 RNA was detected in 51/123 (41.5%) of the samples overall. The presence of SARS-CoV-2 RNA in treated wastewater, irrigation water, field vegetables, and market produce were 75, 37.5, 42.85, and 37.5%, respectively. These results indicate that SARS-CoV-2 RNA is present in food retail and may also suggest that produce can additionally be contaminated with SARS-CoV-2 RNA by agricultural water. This study demonstrates that SARS-CoV-2 RNA was detected in waste and irrigation water, as well as on produce both in field and at retail. However, more evidence is needed to understand if contaminated irrigation water causes SARS-CoV-2 RNA contamination of produce, and if there is a significant public health risk in consuming this produce.

## Introduction

The coronavirus disease 2019 (COVID-19) pandemic began in Wuhan in December 2019 (1). According to the World Health Organization, over 527,806,881 infected individuals and 6,300,785 deaths have been reported. The first confirmed COVID-19 case in Iran was reported on 19 February 2020, and subsequently has spread rapidly in the Iranian population (2).

Transmission of SARS-CoV-2 is primarily thought to occur directly from person to person via respiratory droplets, as well as indirectly through contact with contaminated surfaces (3, 4). Although fecal-oral transmission of SARS-CoV-2 is unlikely to be a major route of transmission, replication in intestinal epithelial cells has been observed (5), with both viral RNA and infectious virus being isolated from patients' feces on the other hand, fecal-oral transmission in other coronaviruses such as SARS-CoV-1 and MERS-CoV is a secondary route of disease transmission, although this transmission has not been proven in SARS-CoV-2 and in it is ambiguity (4, 6–8). Recent studies have shown that SARS-CoV-2 RNA can be detected from the feces and urine of some patients (9–16). Given this, the presence of SARS-CoV-2 RNA in hospital and municipal wastewater has been used as a means of tracking SARS-CoV-2 occurrence in different regions (17–24). SARS-CoV-2 RNA has also been detected in surface waters contaminated by treated or untreated wastewater (25–27).

**Figure 1:**
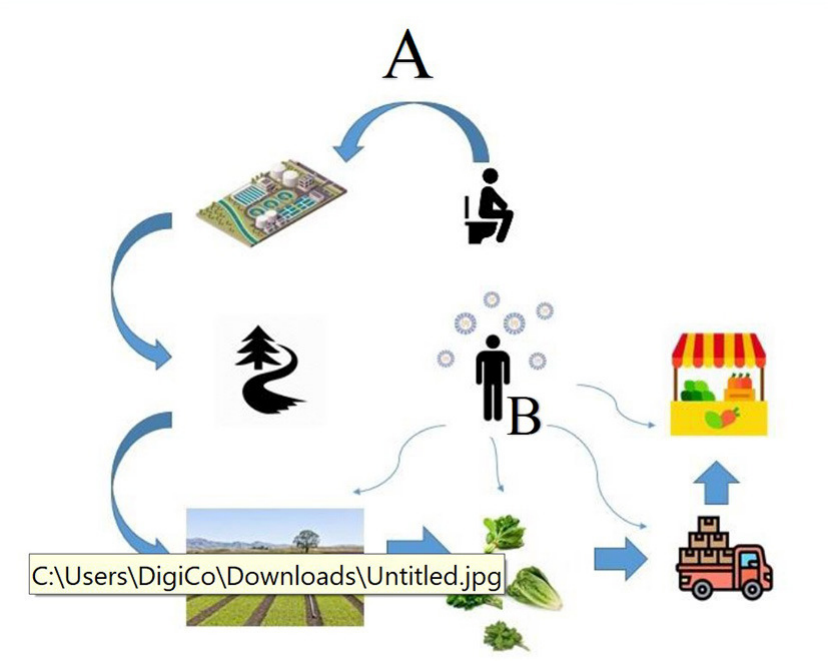
The possible routes of transmission of the SARS-CoV-2 RNA from an infected person to agricultural products. Route A indicates the possible way of contamination in the pre-harvest stages. Route B indicates the possible ways of contamination by food handlers in the post-harvest stages.

Further, the presence of the virus in different communities has been studied by examining raw and treated wastewater, which shows the presence of the virus regardless of the severity of the disease, whether it is symptomatic or not. These studies, known as Wastewater-Based Epidemiology (WBE) studies, were well received after the identification of the virus genome in sewage (28–30). Other studies have also shown the presence of the virus RNA in surface water (25, 27, 31–33) and even in groundwater (33). Thus, contamination of surface and ground water used for irrigation by untreated or treated wastewater presents the possibility that viral contamination of agricultural products could occur. Alternatively, agricultural products that involve a high degree of handling by humans could alternatively be contaminated with SARS-CoV-2 via direct contact or by respiratory droplets at various points during the farm-to-table cycle (33) (Figure 1).

Although fecal-oral transmission of SARS-CoV-2 is not likely, understanding its presence in agricultural water and agricultural products can provide insight into the relative prevalence of SARS-CoV-2 in different communities and food production and retail environments, where SARS-CoV-2 transmission has been noted to occur (34). The potential for contamination of agricultural products by irrigated water that has been contaminated by wastewater exists.

Tehran, is the largest and most populous city in Iran as well as its capital. It is located at the foot of the Alborz Mountains and has a semi-arid climate. Most agricultural production occurs in the south of the city, a region which contains vast agricultural plains near rivers and wells that are used to irrigate these plains. In some cases, irrigation with treated wastewater has been reported. The presence of SARS-CoV-2 RNA in agricultural environments in Iran has not been well characterized, and the purpose of this study was to attempt to better understand the presence of SARS-CoV-2 RNA in select agricultural production environments, markets, and produce in Tehran. It should be noted that due to resource limitations, the presence of infectious SARS-CoV-2 was not investigated, and detection of viral RNA does not directly mean that infectious virus was present in the tested samples.

## Materials and methods

### Background and study design

**Figure 2:**
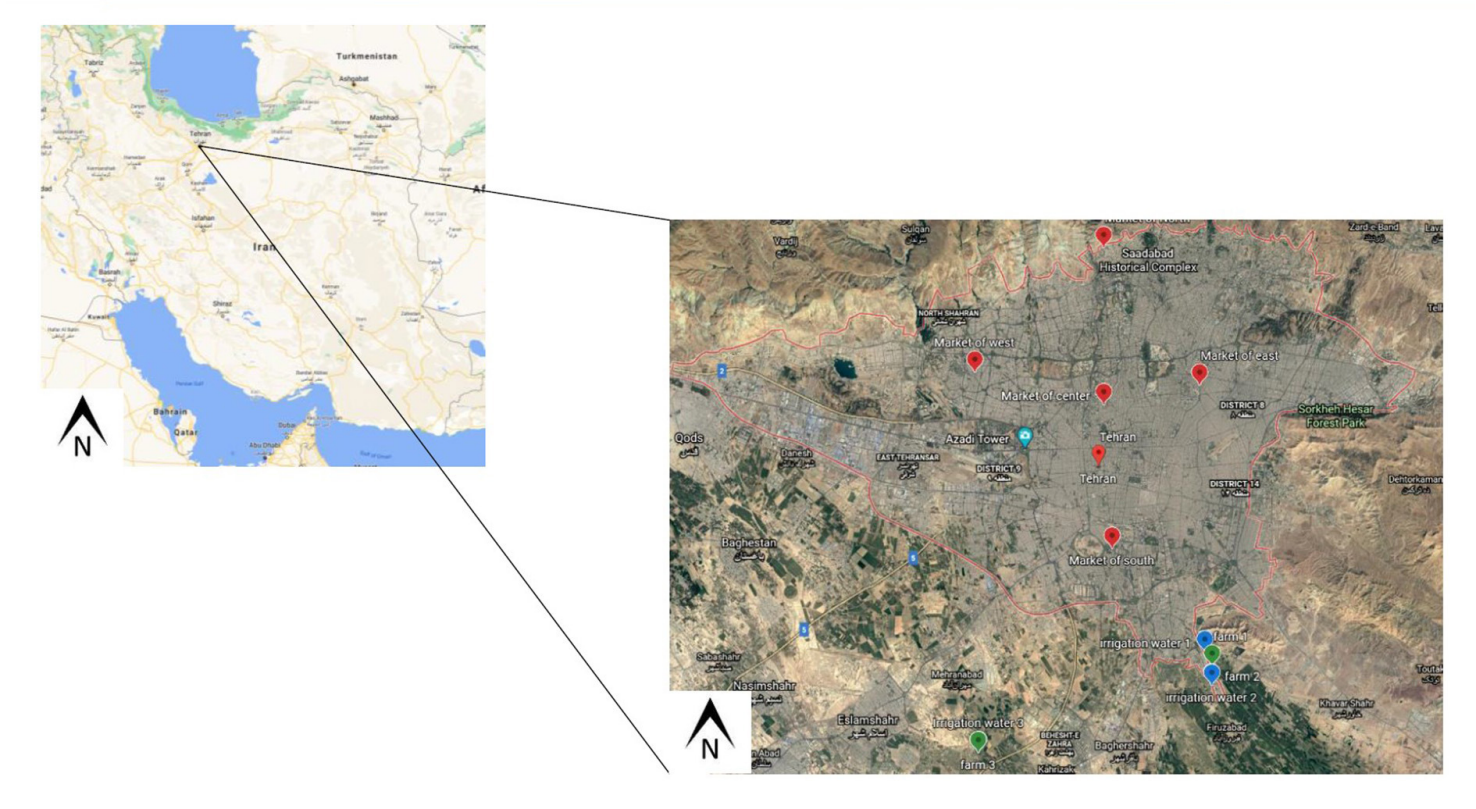
Maps of Tehran, Iran showing the locations where the samples were collected (Locations of fruit and vegetable centers, fields and irrigation water are shown in red, green, and blue, respectively).

The study is designed to investigate the presence of SARS-CoV-2 RNA in treated wastewater, irrigation water, vegetables on farms, and vegetables in markets. Two wastewater treatment plants (WWTPs) in Tehran, three farms in the south of Tehran, and five fruit and vegetable markets were selected for sampling (Figure 2). Farms were selected based on their irrigation water source that was likely to be contaminated via wastewater, and their distance from WWTPs. The size of the farm and the variety of products that were also considered, with similar sizes and products farms selected. Also, regarding fruit and vegetable centers, their geographical location and their size and population have been among the factors involved in their selection.

Fruit and vegetable markets are the primary means that vegetables are purchased in Tehran, with these markets being primarily supplied by farms located in three southern regions of Tehran (Varamin, Kahrizak, and Shahr-e-Rey). Farmers in these areas utilize water from wells, rivers, and urban water canals for irrigation. These water sources have the potential to be contaminated with pathogens by several sources; with treated or untreated wastewater being a prominent example (Figure 2).

### Sampling

From September 2020 to January 2021, 123 samples were collected from in Tehran, Iran. Leafy green samples (*n* = 72) were collected from five fruit and vegetable markets in Tehran, which were geographically divided into five regions (north, south, east, west, and center). In addition, 35 samples were purchased from farms in three important agricultural areas located in the south of Tehran (Varamin, Kahrizak, and Shahr-e-Rey). Furthermore, eight irrigation water samples were collected from a water canal used as an agricultural water supply. Eight treated wastewater samples were taken from a WWTP close to the farms and agricultural water supply (Table 1). All samples, including vegetables (Basil, spinach, cress, lettuce and parsley), irrigation water, and wastewater samples, were stored at in sterile boxes at 4°C, and the viral concentration process was performed within 24 h.

**Table 1:** Distribution of different samples by month and site of sampling.

		**2020**												**2021**			
**Month**		**September**			**October**			**November**			**December**			**January**			**Total**
**Sample**		**Farm**	**Market**	**Total**	**Farm**	**Market**	**Total**	**Farm**	**Market**	**Total**	**Farm**	**Market**	**Total**	**Farm**	**Market**	**Total**	
Wastewater		-	-	2	-	-	0	-	-	2	-	-	2	-	-	2	8
Irrigation water		-	-	2	-	-	2	-	-	2	-	-	1	-	-	1	8
Vegetable	Lettuce	1	3	4	1	2	3	1	4	5	2	4	6	0	4	4	22
	Parsley	2	4	6	2	3	5	2	1	3	2	3	5	1	4	5	24
	Cress	1	2	3	2	2	4	2	5	7	2	3	5	1	1	2	21
	Basil	3	4	7	3	3	6	0	0	0	0	0	0	0	0	0	13
	Spinach	0	1	1	1	5	6	3	5	8	2	5	7	1	4	5	27
Total		7	14	25	9	15	26	8	15	27	8	15	26	3	13	19	123

### Process control virus

The Massachusetts H120 vaccine strain of infectious poultry bronchitis virus was used to evaluate and optimize the concentration method used to isolate SARS-CoV-2 from the agricultural and produce samples. It is a member of the *Coronaviridae* family which is very similar in morphology to SARS-CoV-2. To determine the percentage of virus recovery and validate the concentration method used in this study, 1.5 × 10^6^ TCID_50_ (Median Tissue Culture Infectious Dose) and 1.5 × 10^5^ TCID_50_ of virus were inoculated onto 25 g of lettuce in duplicate. The inoculation process was performed with 50 μl of virus solution, at 10 points with each drop being 5 μl on the surface of lettuce leaves. Virus suspension was then air-dried in a laminar flow hood for 3 h prior to concentration (below). Similarly, 1.5 × 10^5^ TCID50 of the virus was inoculated into 0.5–2 L of agricultural water, then concentration performed.

### Virus concentration

Twenty-five grams of inoculated leafy green samples were cut into 2.5 × 2.5 cm^2^ pieces and placed in a sterile plastic bag. Then, 40 ml of TGBE buffer (100 mM Tris-HCL, 50 mM glycine, 3% beef extract, pH 9.5) was added and the bag was subjected to gentle shaking for 20 min at room temperature. The mixture was passed through a filter (Whatman Grade 41, Fast Ashless filter paper, 150 mm circle, 1441–150) to remove debris particles. All samples were carried out in duplicate. The resulting filtrate was transferred to a centrifuge tube and centrifuged for 30 min at 11,000 × *g* at 4°C. The supernatant was then transferred to a new centrifuge tube to concentrate the eluted viruses with polyethylene glycol (PEG) 6000. PEG precipitation was done by overnight incubation with rocking at 120 rpm at 4°C in the presence of 10% (wt/vol) polyethylene glycol (PEG) 6000 (Sigma-Aldrich, St. Louis, MO) and 0.3 M NaCl at pH 7.2 ± 0.2. After centrifugation at 11,000 × *g* for 30 min at 4°C, pellets were suspended in 200 μL of PBS (pH 7.4) for RNA extraction and stored at −20°C until use (35).

Virus concentration from wastewater and irrigation water samples was performed in a two-step process; electronegative filtration (36) followed by further concentration using polyethylene glycol precipitation (37). In brief, MgCl_2_ was added to the samples to reach a final concentration of 25 mM. Then, 2–3 L irrigation water or about 0.5 L wastewater was filtered using a six-branch filtration system (Sartorious, Goettingen, Germany) with 47 mm nitrocellulose filters with a 0.45 μm pore size (Sartorious). Nitrocellulose filters were then washed with 200 ml of 0.5 mM H_2_SO_4_, and 20 ml of 0.1 mM NaOH to elute the virus from the filter surface and transferred to a solution of 100 μl Tris-EDTA and 50 μl of 100 mM H_2_SO_4._ Afterward, 12.5% PEG 6000 (Sigma-Aldrich, St. Louis, MO) and 2.5% NaCl was used for the second concentration step. At this stage, the samples were placed on the mixer overnight at a temperature of 4°C. The samples were then centrifuged at 11,000 × *g* for 30 min at 4°C. At the end of this step, the supernatant was discarded and precipitate dissolved in 200 μl of PBS. Samples were stored at −20 °C until use.

### RNA extraction

Viral RNA was extracted using the QIAamp RNA mini kit (Qiagen, Germany) using 140 μl of the dissolved pellet suspension above, with 60 μl used for final elution and suspension of RNA per kit protocol. The extracted RNA was stored at −70 °C until use.

### RT-qPCR

To detect the presence of SARS-CoV-2 RNA, a commercial COVID-19 One-Step RT-PCR kit (Pishtaz Teb Diagnostics, Iran) was used containing oligonucleotide primers and probes designed targeting the RdRp and the N regions of the SARS-CoV-2 genome. Furthermore, a primer-probe set targeting human RNase P on a separate channel was used as an internal control. The reaction mix (20μl) consisted of 9 μl resuspended master mix [COVID-19 enzyme mix and RT-qPCR buffer (5x)], and 1μl COVID-19 primer-probe mixture, per kit instructions. Thermal cycling conditions included reverse transcription at 50°C for 15 min, preheating at 95°C for 3 min and 45 cycles of 95°C for 15s and 55°C for 40s, using a Rotor-Gene Q MDx thermal cycler (QIAGEN Hilden, 212 Germany). This Commercial Pishtaz Teb Diagnostics kit is an IVD-approved medical diagnostic kit capable of detecting at least 200 copies/ml of the SARS-CoV-2 genome.

To confirm the detection of SARS-CoV-2 RNA in presumptive positive samples, RT-PCR targeting the ORF-1ab region was also conducted. This amplification was performed using a forward primer (5-TATTATGATTCAATGAGTTATG-3) and reverse primer (5'- GTACTACAGATAGAGACACCAG-3'). Amplified products were electrophoresed on 1.5% agarose gel to visualize DNA bands with expected an product size of 152 bp.

In addition, one PCR product of a positive sample was purified using QIAquick PCR purification kit (Qiagen) and subsequently sequenced using Sanger sequencing in a bi-directional manner with the ABI 3500 automated sequencer from Applied Biosystems (provided by the Genomine Biotech Company, Tehran, Iran). Sequencing results were analyzed using BLAST search with NCBI BLASTN (https://blast.ncbi.nlm.nih.gov).

For control samples, IBV RNA was first converted to cDNA by a high capacity cDNA Reverse Transcription Kit (Thermo Fisher Scientific, USA). A SYBR green qPCR assay containing 12.5 μl of realQ plus 2x master mix green (Ampliqon, Denmark), 5 pmol of forward primer, 5 pmol of reverse primer and 6.5 μl of RNase free water was then used to detect the cDNA. Thermal cycling included preheating at 95 °C for 15 min, and 45 amplification cycles at 95°C for 15 s, 56 °C for 30 s, and 72°C for 30 s (Table 2).

**Table 2:** Primers of PCR assay used in this study.

**Assay**	**Target**	**Primer sequence**	
RT-qPCR SYBR Green	IBV	F	5- GCACAAGGTCGGCTATACG−3
		R	5- GCCATGTTGTCACTGTCTATTG−3
RT-PCR	SARS-CoV-2 (ORF-1ab)	F	5-TATTATGATTCAATGAGTTATG-3
		R	5- GTACTACAGATAGAGACACCAG- 3

IBV RNA was quantified by plotting cycle threshold (CT) to standard curves produced by serial dilution method with RNA extracted from the Massachusetts H120 vaccine strain. The standard curve showed a linear dynamic range from 10^2^ to 10^6^ copies for IBV (y = −3.710x + 41.110, R^2^ = 0.99).

### Quality control

On a regular basis in each sampling period, 2 samples were spiked with 1.5 × 10^6^ TCID_50_ IBV as a positive control sample and one sample which was thoroughly washed and exposed to ultraviolet light for 20 min as a negative control sample to ensure the absence of false-positive (possible cross-contamination) and false negative (possible recovery failure) results. Further, a positive sample and negative sample were tested for quality control along with all water samples in each sampling period. Both RT-qPCR assays included negative (nuclease-free water) and positive amplification controls (For IBV, RNA extracted from the Massachusetts H120 vaccine strain was used, and for SARS-CoV-2, a plasmid designed by the SARS-CoV-2 RT-qPCR kit (Pishtaz Teb Diagnostics, Iran) with target RNA fragments, RdRp and N, were used as a positive control).

## Results

The method of detecting SARS-CoV-2 RNA from the vegetable samples as well as concentrating samples of irrigation water and wastewater treated by IBV inoculation was tested. On average, IBV recovery in vegetable samples was calculated to be about 22.4 ± 9% for direct samples and 16.8 ± 3% for 10-fold dilutions. Furthermore, in the samples of irrigated water and treated wastewater, 21.1 ± 3.2% and 11.8 ± 1.8% recovery were observed, respectively.

**Figure 3:**
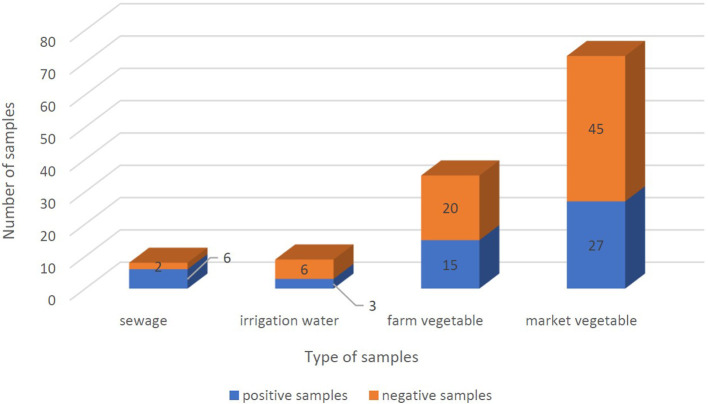
Positivity of SARS-CoV-2 RNA frequency in sewage, irrigation water, farm vegetables, and market vegetables samples. Positive samples and negative samples are shown in blue and orange, respectively. And the whole columns show the total number of samples.

**Figure 4:**
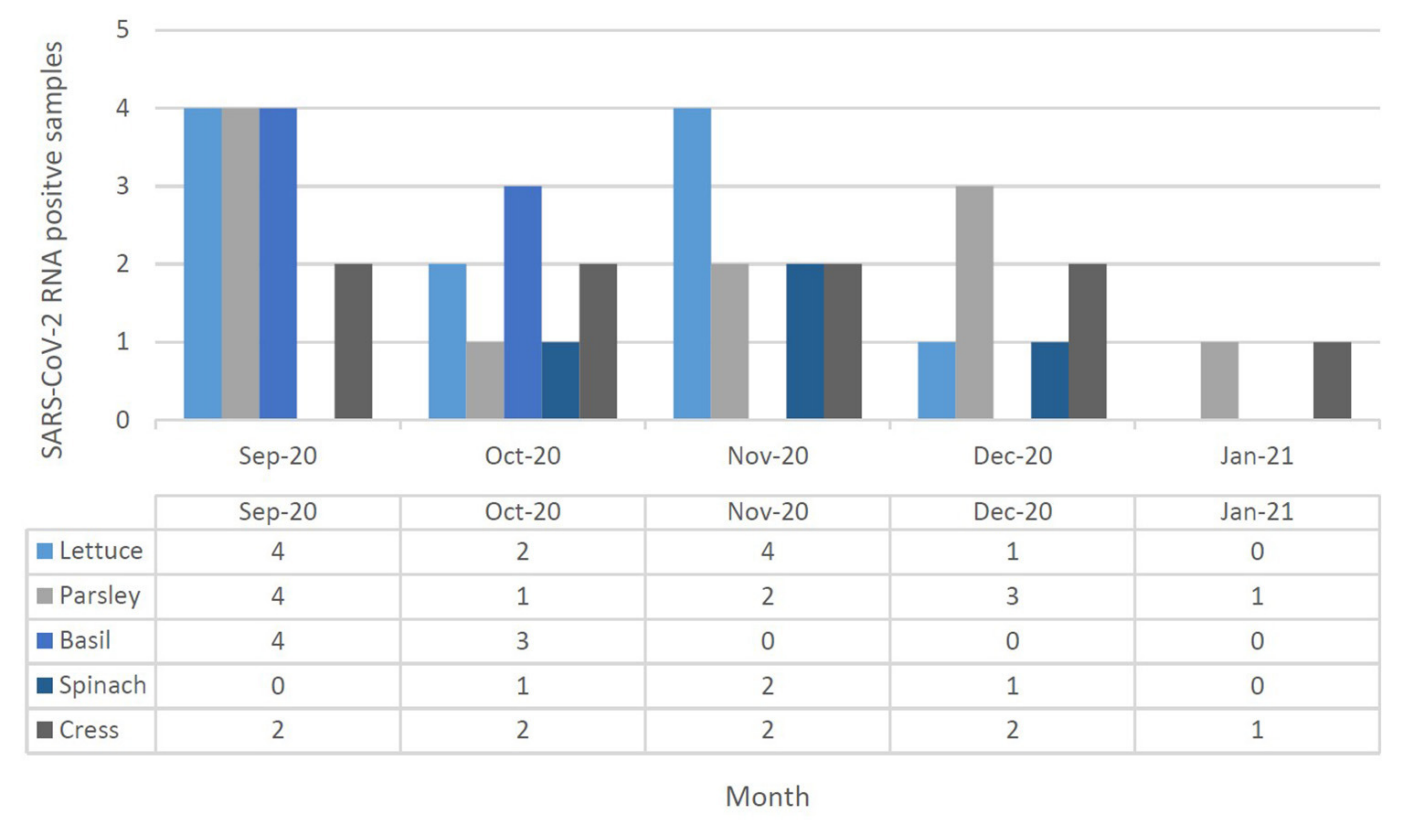
SARS-CoV-2 RNA presence in leafy green vegetable (lettuce, parsley, basil, spinach, and cress) collected in September 2020 to January 2021 at Tehran, Iran. Different leafy green vegetables were shown in different color in each month, separately.

From September 2020 to January 2021, 123 samples from 2 WWTPs, 3 irrigation sites, and 3 farms, and 5 fruit and vegetable markets in Tehran were tested twice for the presence of SARS-CoV-2 RNA. Samples were considered positive if at least one target gene in one of the duplicate samples had a CT below 40. SARS-CoV-2 RNA was detected in 51 of the 123 (41.5%) samples, overall. SARS-CoV-2 RNA was detected in 75% (6/8), 37.5% (3/8), and 39.25% (42/107) of treated wastewater, irrigation water, and vegetable samples, respectively. Leafy greens purchased from the farms and markets were positive for SARS-CoV-2 RNA in 42.85% (15/35) and 37.5% (27/72) of samples, respectively (Figures 3, 4). In all duplicate samples, the RdRp gene was examined, and its CT ranged from 30.54 to 39.22. RdRp gene compared to the N gene, a better range of CT (29.5–36.59) was also observed compared to the N gene.

## Discussion

This study evaluated the presence of SARS-CoV-2 RNA in agricultural and food samples in Iran, as markets and agricultural environments have been known to be sites of SARS-CoV-2 outbreaks. The results of this work suggest that SARS-CoV-2 RNA was present in a notable percentage of agricultural water and produce samples, as well as wastewater samples. These samples could have been contaminated at pre- or post-harvest stages. At the pre-harvest stage, the crops can be contaminated at different phases of growth by contaminated fertilizers, sewage, or irrigation water; however, the specific means by which contamination occurred was not determined in this work. One report suggests that treated wastewater caused SARS-CoV-2 contamination of irrigation water in the south of Tehran, and this is potentially an explanation for the observed contamination of irrigation water reported here, (37.5% of irrigation water samples contained SARS-CoV-2 RNA) but more work would need to be conducted to confirm this. In the post-harvest stage, person-to-person or food handler contamination in markets are could be sources of contamination, though the specific means of contamination of produce in the markets was not determined in this work. Infected food handlers can be important sources of transmitting the virus products at the time of harvesting, packaging, transmission, classification, and selling (38). Additionally, post-harvest contamination could occur through washing with contaminated water. SARS-CoV-2 RNA has been detected in municipal wastewater in several countries such as Australia, Japan, Italy, Spain, USA, Germany, and others, including Iran (17–23). Following these cases, a study was conducted in Iran on treated wastewater that suggested the effluent of treatment plants was discharged into surface water, contaminating it with SARS-CoV-2 RNA (24). Similar work in Italy (25) and Japan (26) failed to detect SARS-CoV-2 RNA in surface water, while it was observed in a study in Ecuador (27). Subsequently, a study in India reported the presence of SARS-CoV-2 RNA in 56% of river samples and 53% of lake samples. In Serbia, 50% of collected river samples were found to contain SARS-CoV-2 RNA. In a recent study in Nepal, 47 and 69% of treated wastewater and river water contained SARS-CoV-2 RNA, respectively. In the present study, SARS-CoV-2 RNA was found in 75 and 37.5% of treated wastewater and irrigation water, respectively. SARS-CoV-2 RNA can directly contaminate surface water by dumping of treated or incompletely treated wastewater, or in some cases, raw wastewater, and this water may have been used to irrigate Tehran vegetables; however, further confirmatory work to determine the specific routes of contamination is needed. To our knowledge, this is the first systematic investigation of the presence of SARS-CoV-2 RNA on vegetables and their irrigation water in Iran.

Our results demonstrate that the presence of SARS-CoV-2 RNA in Tehran is not limited to surface water, as SARS-CoV-2 RNA was also detected in vegetables on farms and in markets; suggesting that SARS-CoV-2 RNA is prevalent in agricultural production environments, produce, and in markets in Tehran. Two agricultural areas in the south of Tehran were studied, in one (Varamin and Shahr-e-rey area) the field was irrigated with surface water, while the other one (Kahrizak area) was irrigated with well water. Water samples taken from these two areas show surface water pollution as opposed to well water, as we failed to detect SARS-CoV-2 RNA in the well water source tested as well as the vegetables on which it was applied. However, SARS-CoV-2 RNA was detected in the surface waters of Rey city, and like their water, 63.15% of the vegetables tested were observed to contain SARS-CoV-2 RNA.

In fruit and vegetable markets, the different levels of contamination were observed. These markets were in different parts of Tehran, and differed in client volume, the volume of products offered, and the number of employees; all of which have potential to influence the level of contamination observed. For example, the southern fruit and vegetable markets are one of the largest fruit and vegetable centers in Tehran, with a larger number of customers and staff, as well as a high range of products, which in turn can cause less observance of personal and social hygiene. As expected, the amount of vegetable contamination seen in these markets was higher than the others, suggesting such contamination may be an indicator of more widespread infection in the markets (Table 3). On the other hand, the results show that the presence of SARS-CoV-2 RNA in the studied vegetables seems to be different, which requires further investigation in the future (Table 4). This study is the first to indicate contamination of vegetables with SARS-CoV-2 RNA in Iran. Based on results, there is a good amount of value in understanding the presence of SARS-CoV-2 in agricultural production and retail environments, and that irrigation water might be one route through which SARS-CoV-2 can be introduced into these environments. This is particularly relevant to agricultural workers, as the irrigation water itself may be aerosolized in its application/handling, and the produce may also have potential to serve as fomites to agricultural and retail workers who handle the produce. The hypothesis that food packages can become contaminated with SARS-CoV-2 and thus cause fomite transmission of SARS-CoV-2 in humans was suggested by Liu et al. (39) on frozen Cod fish packages. However, more research is needed to determine the relative risk such presence of SARS-CoV-2 in these environments poses to agricultural and retail workers.

**Table 3:** Geographical prevalence of SARS-CoV-2 RNA in fresh produce from food and vegetable center of Tehran.

**Site No**.	**Food and vegetable center**	**Geographical location**	**Positivity of SARS-CoV-2 RNA**
1	Velenjak	North	6
2	Sayad Shirazi	East	5
3	Jalal Alahmad	Center	3
4	Jannat Abad	West	4
5	Bahman	South	10

**Table 4:** Prevalence of SARS-CoV-2 RNA in different type of vegetable samples that was selected from farms and markets.

**Type of sample**	**Farm**		**Market**		**Total**	
	**No. of samples**	**No. of positivity**	**No. of samples**	**No. of positivity**	**No. of samples**	**No. of positivity**
Lettuce	5	2	17	10	22	12
Parsley	9	5	15	6	24	11
Cress	8	4	13	5	21	9
Basil	6	3	7	4	13	7
Spinach	7	1	20	3	27	4

Further, it should be noted that all of the data reported here involves detection of SARS-CoV-2 RNA, and not infectious virus. This is an important distinction, as viral RNA can persist in the environment notably longer than infectious virus. Future work is needed to elucidate the degree to which infectious SARS-CoV-2 occurs in these agricultural and retail settings in Iran; and this would be crucial to better gauge the degree to which the presence of SARS-CoV-2 in these environments poses a threat to public health.

In addition to the fact that only viral RNA was detected and not infectious virus, there were a number of other limitations in this study. (1) The number of farms was limited due to a lack of broader cooperation among farm owners. (2) Vegetables were not available in all sampling months due to their seasonal cultivation, and the potential influence of seasonality could confound results. (3) Specifically, only farms that utilize irrigation water were tested and not farms that did not utilize irrigation water. The degree to which SARS-CoV-2 RNA occurs in those types of farms should be determined in subsequent work. (4) Specific lockdown and other public health measures also limited the degree to which sampling could occur.

## Conclusion

In summary, this is the first study to report the presence of SARS-CoV-2 RNA in irrigation water samples and on vegetable surfaces in Iran. From 123 samples, SARS-CoV-2 RNA was detected in 51 of them. Among the 42 positive vegetable samples, 35.7% and 64.3% were tested in farms and markets, respectively. Although the specific routes of contamination of these samples was not determined, these results suggest a high prevalence of SARS-CoV-2 RNA in agricultural and retail settings in Tehran, and suggest that such prevalence can be observed across multiple ends of the produce production chain. However, more studies should be conducted in future to examine the degree to which infectious SARS-CoV-2 occurs in these settings, and if so, where significant points of contamination occur.

## Data availability statement

The raw data supporting the conclusions of this article will be made available by the authors, without undue reservation.

## Author contributions

MR: formal analysis, methodology, investigation, writing—original draft, and supervision. SM: conceptualization, formal analysis, methodology, validation, writing—review and editing, and project administration. SH: conceptualization and writing—review and editing. MT: formal analysis, methodology, software, investigation, and writing—original draft. MS and SK: investigation and formal analysis. HA: resources, writing—review and editing, and funding acquisition. MM: writing—review and editing. MZ: conceptualization, resources, writing—review and editing, and funding acquisition. All authors contributed to the article and approved the submitted version.

## Funding

This present study was funded by the Research Institute for Gastroenterology and Liver Diseases, Shahid Beheshti University of Medical Sciences, Tehran, Iran (Grant number: IR-RIGLD-1157).

## Conflict of interest

The authors declare that the research was conducted in the absence of any commercial or financial relationships that could be construed as a potential conflict of interest.

## Publisher's note

All claims expressed in this article are solely those of the authors and do not necessarily represent those of their affiliated organizations, or those of the publisher, the editors and the reviewers. Any product that may be evaluated in this article, or claim that may be made by its manufacturer, is not guaranteed or endorsed by the publisher.
